# Understanding Emergency Room Visits for Nontraumatic Oral Health Conditions in a Hospital Serving Rural Appalachia: Dental Informatics Study

**DOI:** 10.2196/31433

**Published:** 2022-12-23

**Authors:** Raj K Khanna, Alfred A Cecchetti, Niharika Bhardwaj, Bobbi Steele Muto, Usha Murughiyan

**Affiliations:** 1 Department of Dentistry, Oral & Maxillofacial Surgery Joan C Edwards School of Medicine Marshall University Huntington, WV United States; 2 Department of Clinical and Translational Science Joan C Edwards School of Medicine Marshall University Huntington, WV United States

**Keywords:** dental informatics, visualization, nontraumatic dental care, emergency room, cost, utilization, economic impact

## Abstract

**Background:**

In the Appalachian region, a variety of factors will impact the ability of patients to maintain good oral health, which is essential for overall health and well-being. Oral health issues have led to high costs within the Appalachian hospital system. Dental informatics examines preventable dental conditions to understand the problem and suggest cost containment.

**Objective:**

We aimed to demonstrate the value of dental informatics in dental health care in rural Appalachia by presenting a research study that measured emergency room (ER) use for nontraumatic dental conditions (NTDCs) and the associated economic impact in a hospital system that primarily serves rural Appalachia.

**Methods:**

The Appalachian Clinical and Translational Science Institute’s oral health data mart with relevant data on patients (n=8372) with ER encounters for NTDC between 2010 and 2018 was created using Appalachian Clinical and Translational Science Institute’s research data warehouse. Exploratory analysis was then performed by developing an interactive Tableau dashboard. Dental Informatics provided the platform whereby the overall burden of these encounters, along with disparities in burden by age groups, gender, and primary payer, was assessed.

**Results:**

Dental informatics was essential in understanding the overall problem and provided an interactive and easily comprehensible visualization of the situation. We found that ER visits for NTDCs declined by 40% from 2010 to 2018, but a higher percentage of visits required inpatient care and surgical intervention.

**Conclusions:**

Dental informatics can provide the necessary tools and support to health care systems and state health departments across Appalachia to address serious dental problems. In this case, informatics helped identify that although inappropriate ER use for NTDCs diminished due to ER diversion efforts, they remain a significant burden. Through its visualization and data extraction techniques, dental informatics can help produce policy changes by promoting models that improve access to preventive care.

## Introduction

### Overview

Oral health is critical to overall health and well-being [1]. However, various factors impact the ability to maintain oral health, such as the availability of excellent and accessible preventive dental care, socioeconomic status, age, location, race, ethnicity, and health behaviors [1]. Poor oral health or the absence of affordable regular dental care negatively affects health (associated with diabetes, heart disease, stroke, increased admission risk, and adverse pregnancy outcomes) and increases health care costs [2-7]. An emerging field, dental informatics, which involves the application of informatics to dental science, is increasingly used to solve problems in dental practice, education, training, and research [8-10].

Appalachia, a region with a largely rural population, spreads over 420 counties in 13 states. Rural Appalachia is medically underserved to the extent that there is an average of only 4 dentists per 100,000 individuals, compared to the United States’ average of nearly 61 dentists per 100,000 [11]. West Virginia (WV) is the only state of the 13 states entirely in Rural Appalachia that is underserved and hit hard economically. WV leads the nation in edentulism, which afflicts 36% of adults, and reports indicate that only 61% of adults visited a dentist in the past year [12]. However, due to economic constraints, most health systems in Appalachia do not have the technological resources needed to investigate and solve the oral health issues of this population.

There are several indicators of oral health in a community. One such indicator is a visit to an emergency room (ER) for a preventable dental condition. When people don’t have access to preventive dental care for problems like gum disease and tooth decay, treatable dental issues become a much bigger problem, often causing excruciating pain, leading people to seek care in an emergency department. Data show that ERs are the first and last resort for many low-income adults nationwide to obtain emergency care for preventable dental conditions. ERs are a significant part of the health care system that should be used for emergency health care needs and should not be a place for routine dental care. Further, these settings are ineffective for treating dental problems [13]. Over the past few years, dental care has increasingly shifted from dental offices to emergency departments, with a more significant portion of dental visits occurring in EDs [14]. ER use for dental problems is on the rise, and most visits are for nontraumatic dental conditions (NTDCs) [15,16]. It is harder to get good dental care in Appalachia, especially in WV, which encompasses some of the country’s poorest and most remote communities. Compounding decreased availability of dental care and lower socioeconomic status is illiteracy. These factors contribute to the increased number of visits to the ER.

The use and financial impact of dental-related trips to medical settings, such as ERs and urgent care sites in the Huntington hospital system’s catchment area in WV, has not been documented. We used dental informatics to develop a synopsis of dental visits to ER for preventable dental conditions in southern WV.

This paper highlights how dental informatics was used to examine preventable dental conditions in the Appalachian region of WV and suggests cost containment.

### Literature Review

Dental informatics, a comparatively juvenile field [17], can be used to understand a wide variety of problems within dentistry but is not widely used [18] and historically may not be widely understood [19]. One of the difficulties with global acceptance is that dental practitioners may lack computer knowledge, creating a hurdle for introducing dental informatics [20]. e-Communities within dental societies may overcome these difficulties by bringing dental professionals together at a resource hub and allowing them to share information [21].

Dental informatics can assist in patient care [22] with new methods grounded in machine learning that can be used in the detection of teeth, caries, restored teeth, dental crowns, dental implants, prostheses for dentofacial deformities [23], oral pathology [24], oral cancer screening [25], and endodontic treatment[26]. Dental informatics has phenomenal potential in evaluating public health initiatives, and it could be used for the assessment of health goals achievements [26] as well as in education [27]; however, it has functional limitations resulting from medical and dental patient care not being stored in the same system. Dental practices typically silo their health care delivery systems, hindering easy access to institutional analysis [10].

Integrating dental informatics into health information systems provides an effective service. These combined systems will collect data from population-based surveys, disease surveillance systems, hospital information systems, and family health surveys and provide access to whole-patient patterns that can improve patient dental services [28]. Providing examples of how integrated dental informatics can focus on problems and provide solutions is expected to lead to more adaptive, patient-focused, and efficient dental care with educational advantages in training [29].

To understand the more enormous challenges within dentistry, especially concerning health in rural areas [30], the dental informatics system will require extensive collections of patient information [31], with both dental and medical information. Dental informatics can benefit the Appalachian region, but institutional costs are always of concern [32]. Hence, examples of how dental informatics could identify and initiate cost containment are of high value.

## Methods

### Procedures

The Appalachian Clinical and Translational Science Institute (ACTSI) Division of Clinical Informatics has a functional multi-institutional clinical research data warehouse (CRDW) containing more than 12 years of billing and electronic medical record data. The CRDW consists of relational tables, dimensions, and fact tables (Online Analytical Processing cube) that store multi-institutional medical information and provide data for operational and analytical model development (machine learning). It contains structured electronic health record data (eg, vitals, medication, procedure, and diagnosis), non–electronic health record survey data, and unstructured (text) information received from Marshall Health practice plan, Cabell-Huntington Hospital, and Marshall University Joan C Edwards School of Medicine’s Edwards Comprehensive Cancer Center. It uses the technological tools of information science (eg, computer workstations, mobile smartphones, interactive visualization, programming, and machine learning ) to build a platform that can gather, analyze, and present information to address, in this case, oral health needs [33].

For this retrospective longitudinal study, we used the oral health data mart that was developed using the ACTSI’s CRDW to understand and improve the dental health of the population in the area. Relevant clinical and financial data from the data mart over 9 years (2010-2018) were extracted, verified, and analyzed. ER encounters for preventable dental conditions were identified using the primary diagnosis codes (International Classification of Diseases, Ninth Revision, Clinical Modification; and International Classification of Diseases, Tenth Revision, Clinical Modification). We calculated the overall burden of dental-related ER visits for avoidable conditions. Disparities in their burden regarding demographic variables, such as patient’s age, gender, and primary insurance for the visit, were also examined. Further, patterns and trends in ER use for such visits and the associated charges were studied.

### Ethical Considerations

The retrospective research study was conducted with the best intent and information obtained from the CRDW. This study was approved by the institutional review board of Marshall University, Huntington, WV (1069363-1).

## Results

**Figure 1:**
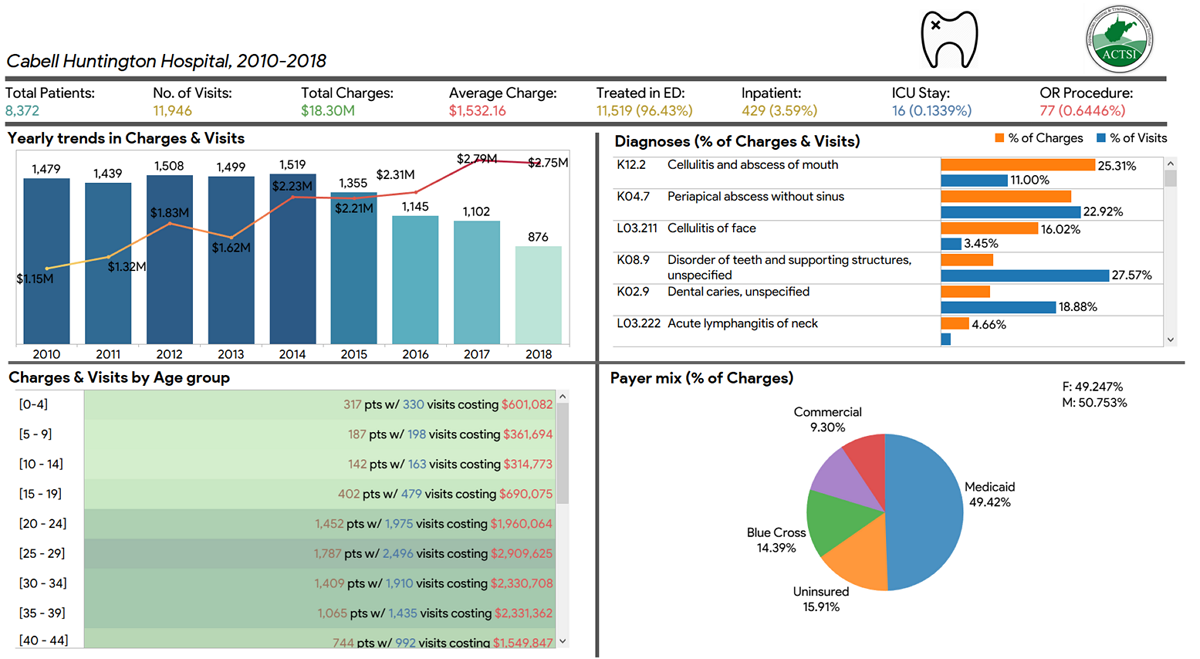
Tableau dashboard displaying patterns and trends in charges and visits for nontraumatic dental conditions to the emergency department at Cabell Huntington Hospital between 2010 and 2018. ED: emergency department; ICU: intensive care unit; OR: operating room.

Transact-SQL coding found that 8372 patients made 11,946 visits to the ER for a nontraumatic dental condition and generated US $18,303,173 worth of hospital charges over 9 years (Figure 1). Of these 8372 patients, 4123 (49.25%) were female, and 4249 (50.75%) were male, with mean ages of 32.43 (SD 0.20) and 32.37 (SD 0.198) years, respectively.

Using the oral health data mart, we found that although the number of visits decreased yearly, the charges quadrupled, with average costs per visit increasing from US $776.64 in 2010 to US $3136.79 in 2018. Additionally, the percentage of visits resulting in an inpatient admission or requiring a surgical intervention rose yearly from 2010 to 2018. Most of these visits were for teeth and supporting structure disorders and periapical abscesses without sinus. Meanwhile, the visits for cellulitis and mouth abscesses were the most expensive. This information was presented using a Tableau dashboard, which provided an interactive display that was informative but also simple to understand (Figure 1).

Medicaid was the primary payer for most of these visits in 2018 compared to a third of the payments in 2010. Of all the age groups, adults between the ages of 25 and 29 years had the highest visits and charges. It has also been noted that there was no distinct gender predisposition in the number of visits and charges accrued. However, it has been observed that female patients’ ER visits were less for NTDCs compared to ER visits by male patients. Other predictors of an ER visit for NTDCs could be the age factor and the individuals’ insurance status. It has been observed that individuals between the ages of 25 and 29 years with no insurance coverage had more ER visits. A similar trend was observed regarding the insurance status, as ER visits by uninsured patients were higher than those by the insured patients. The type of insurance also played a role in the ER visits, as it has been noted that Medicaid-insured patients were more likely to be in ER for dental problems compared to Medicare-insured patients.

**Figure 2:**
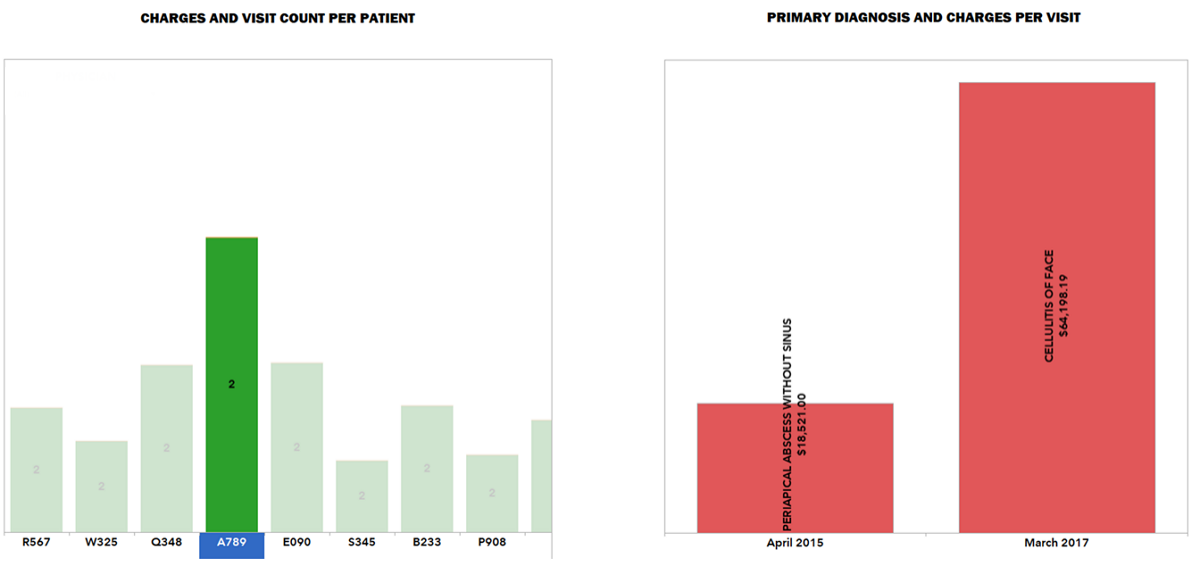
Tableau drill-down. Patient-centered view showing all ER visits with corresponding primary diagnosis and charges for the selected patient.

Using interactive visualization tools, we were able to drill down to the patient level, which showed that patients returned to the ER for NTDCs multiple times. An example of such visits by one such patient, along with the primary diagnosis and charges for the visits, is shown in Tableau-derived Figure 2.

## Discussion

### Principal Findings

Dental informatics combines technological tools with information science to hasten improvements in dental practice, research, education, and management [21,24,34]. In this paper, we examined how dental informatics was used to identify the vast resources spent on dental visits to the ER for NTDCs and how it was used to help formulate a plan to channel these resources toward preventive care, such as setting up mobile dental clinics or free oral health care checkups for the homeless to reduce ER dental visits.

Using dental informatics, we found that ER dental visits for preventable conditions posed a significant burden of more than US $18.3 million. Further, despite a decelerating trend in the number of visits from 2010 to 2018, the average charges per patient per visit have increased. An accelerating trend has been traced in the percentage of visits that required hospital admissions and dental procedures for the same tenure. Dental informatics was instrumental in demonstrating that patients who visited the ER for NTDCs tended to be sicker every year, accruing more charges per visit. We speculate that this occurred due to the institution of services, such as a mobile dental clinic and residency programs, leading to diverting patients with less severe dental conditions from the ER settings.

### Comparisons

In the past, several programs promoting these types of services have successfully reduced ER use for NTDCs [35]. Despite these measures, ER visits for NTDCs continue to be a substantial strain on the health care system, with emergency services provided in the ER being the only dental coverage available to many patients in WV and surrounding regions [36]. Further, ERs are not well suited for treating dental conditions and promoting oral hygiene habits to prevent the problem from recurring; patients tended to come multiple times for similar dental issues, which we noticed in the patient-centered view of our interactive dashboard. We continue to use dental informatics to explore these areas of high interest and concern.

### Limitations

This retrospective study used available data from 2010 to 2018 and consisted of those patients seen within the Marshall Health practice plan, Cabell-Huntington Hospital, and Edwards Comprehensive Cancer Center. This study did not include other outside hospital systems within the local area.

### Conclusions

Dental informatics tools and approaches improve the dental practice’s understanding and help assess dental services’ economic burden [8]. Using dental informatics, we found that emergency department dental visits remain a significant and costly public health problem for vulnerable individuals. Institution of ER dental diversion services may lead to lower costs, which is more effective and can provide more appropriate care. However, they do not entirely address the issue, as there is a need to expand existing medical coverage, including coverage for preventive or restorative dental care. This will require buy-in from payers, an area where visualization tools can play an important part. But providing dental coverage alone might not be enough to reduce dental ER visits if patients do not have access to dental providers [37]. Thus, policy changes that direct efforts enabling dental coverage and access to preventive oral care, resulting in care quality and cost benefits, are necessary. These changes could save millions annually (US $2.75 million in avoidable charges in 2018) in the southern WV region alone. Dental informatics played an especially significant role in this Appalachia area study by providing accurate information in easy-to-understand presentations. This is particularly important in poorer rural areas, such as Appalachia, where multiple medical issues compete for scarce funding and attention.

Appalachian hospitals are especially concerned about costs with affordability, equity, and nonhealth benefits, factoring into decisions about health spending [38]. It is essential to present examples of how an Appalachian hospital’s dental group, using dental informatics, could find and suggest solutions to a problem and guide decisions about where to spend limited resources that respond to the population’s most significant health needs.

## Abbreviations

ACTSI: Appalachian Clinical and Translational Science Institute
CRDW: clinical research data warehouse
ER: emergency room
NTDC: nontraumatic dental condition
